# Revised complete genome sequences of *Limosilactobacillus reuteri* DSM 20016^T^ and ATCC PTA-6475 and confirmation of an intragenic macrosatellite in adhesin gene *cmbA*

**DOI:** 10.1128/spectrum.04005-25

**Published:** 2026-06-15

**Authors:** Mohamed R. Kady, Robert A. Britton

**Affiliations:** 1 Department of Molecular Virology and Microbiology, Baylor College of Medicine 189531, Houston, Texas, USA; 2 Medical Scientist Training Program, Baylor College of Medicine 3989, Houston, Texas, USA; 3 Alkek Center for Metagenomics and Microbiome Research, Baylor College of Medicine 3989, Houston, Texas, USA; 4 Dan L. Duncan Comprehensive Cancer Center, Baylor College of Medicine 3989, Houston, Texas, USA; University of Nevada Reno, Reno, Nevada, USA

**Keywords:** *Limosilactobacillus reuteri*, genomics, DNA sequencing, tandem repeat sequences, polymerase chain reaction

## Abstract

Cell and mucus binding protein A (CmbA) is a cell-wall-anchored adhesin common to human isolates of *Limosilactobacillus reuteri*, which governs mucosal adhesion *in vitro*. Recent attempts to sequence *cmbA* in different *L. reuteri* strains revealed significant genomic inconsistencies with the publicly available closed genome sequences, especially with that of the *L. reuteri* type strain, DSM 20016^T^. We report here a revised closed genome sequence for DSM 20016^T^ and a closed genome sequence for the closely related *L. reuteri* ATCC PTA-6475 (MM4-1A). Hybrid long- and short-read sequencing demonstrated that two genomic regions totaling 40 kbp, previously thought to be absent in DSM 20016^T^, were in fact intact. The *cmbA* gene, present in one of these regions, is the longest predicted gene in both genomes and was confirmed to contain an intragenic tandem repeat region. In DSM 20016^T^, the region consists of 11 identical ~290 bp tandem direct repeats totaling 3.2 kbp, while ATCC PTA-6475 has 8 repeats totaling 2.3 kbp. This macrosatellite posed a challenge to PCR-based approaches to confirm the length of the gene. Polyacrylamide gel electrophoresis of cell wall extracts from ATCC PTA-6475 showed a ~160 kDa band, which was absent from a *cmbA*-knockout strain, consistent with the expected size based on whole-genome sequencing and confirmed by mass spectrometry to be CmbA. Overall, we present refined publicly available genome sequences for two frequently studied *L. reuteri* strains and validate the length of a large gene with a conspicuously high number of identical tandem repeats.

**IMPORTANCE:**

Studies comparing bacterial genomes and routine cloning work often implicitly assume that the closed genome sequences available from public databases are accurate. However, as technologies improve and we gain new data, inconsistencies can arise which prompt the resequencing of strains, sometimes with surprising results. We show here that a significant sequencing assembly artifact led to a large gap in the publicly available closed genome of the *Limosilactobacillus reuteri* type strain which has remained uncorrected for nearly two decades, despite a vast body of *L. reuteri* work over that time. Another region contained a large stretch of repetitive intragenic DNA that still posed a challenge to modern PCR techniques. Therefore, in addition to being useful to *L. reuteri* biologists, this work serves as an important reminder of the intrinsically experimental nature of sequencing data; it usually pays to resequence early and often.

## OBSERVATION

*Limosilactobacillus reuteri* is a gram-positive heterofermentative lactic acid bacterium found in the gastrointestinal tract of many vertebrates. The type strain, F 275^T^, was deposited in 1972 with what is now the German Collection of Microorganisms and Cell Cultures (DSMZ) as DSM 20016^T^ (1). In 2007, a closed genome sequence for DSM 20016^T^ was published (RefSeq accession number NC_009513) (2), followed by one for JCM 1112^T^ (NC_010609), a subculture of DSM 20016^T^ deposited in 1982 with the Japan Collection of Microorganisms (JCM) (3). Unexpectedly, the JCM 1112^T^ sequence contained two chromosomal regions of 30.2 and 8.4 kbp absent from the DSM 20016^T^ sequence (2, 3). As the regions are flanked by mobile genetic elements, these discrepancies were interpreted as genomic deletions which occurred during laboratory passage of DSM 20016^T^ (2).

The 30.2 kbp region contains genes for nitrate reductase, molybdopterin biosynthesis, iron transport, and the cell-wall-anchored adhesin CmbA/Lar_0958 (3–5). The 8.4 kbp region encodes four glycolytic enzymes, including the only copy of glyceraldehyde-3-phosphate dehydrogenase (GAPDH) present in the DSM 20016^T^ genome (6). The conspicuous absence of such a critical gene for glycolysis, combined with a newer DSM 20016^T^ draft genome sequence from 2015 (NZ_AZDD01) that *does* contain the two regions (7) and several independent reports (5, 6, 8), led us to hypothesize that at least part of the two “missing” regions were intact in DSM 20016^T^ after all. As type strain genomes serve as important reference points for comparative genomics, we thought it pertinent to resequence DSM 20016^T^ with hybrid long-read Oxford Nanopore Technology (ONT) and short-read Illumina sequencing to clarify the status of these regions. We also report the first closed genome sequence for the closely related strain ATCC PTA-6475 (MM4-1A) (9), for which only a draft assembly was previously available (NZ_ACGX02) (10).

**Fig 1:**
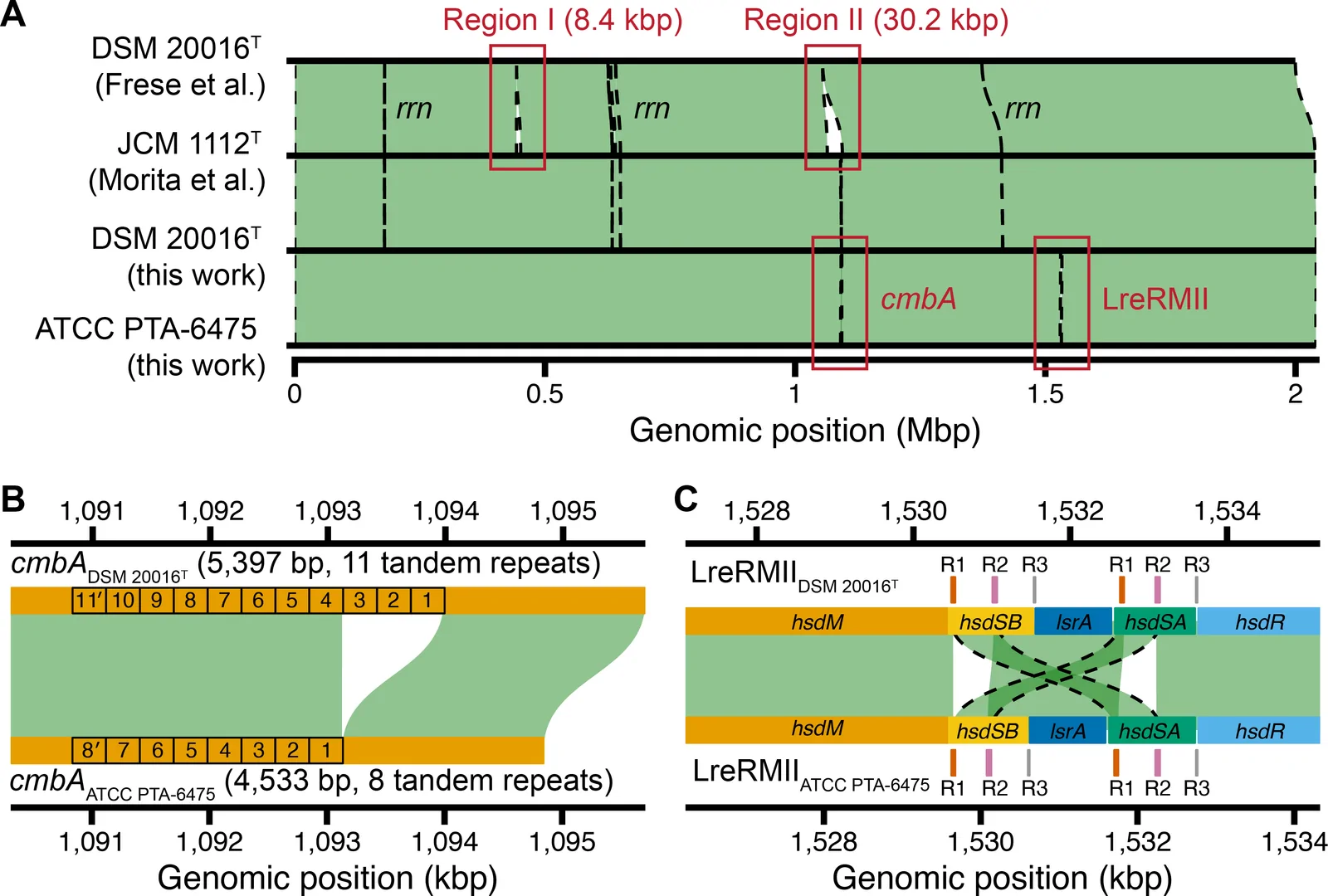
Highlighted sequence conflicts between previous *L. reuteri* sequencing attempts and this work. (**A**) Whole-genome pairwise alignments (≥99.7% identity, ≥4,500 bp), with dotted lines indicating breakpoints. “*rrn*” denotes breakpoints arising in and around rRNA operons; “Region I” and “Region II” refer to the regions unique to JCM 1112^T^ compared to DSM 20016^T^ as previously described (3). (**B**) Pairwise alignment of *cmbA* between the DSM 20016^T^ and ATCC PTA-6475 sequences generated in this work (100% identity), showing the difference in repeat number between strains. The last repeat, denoted by a prime symbol, lacks the final 3 bp (285 bp) of the previous repeats (288 bp). (**C**) Pairwise alignment of the LreRMII locus between this work’s DSM 20016^T^ and ATCC PTA-6475 sequences (>99% identity), showing the reciprocal translocation of *hsdS* domains. Gene nomenclature is as previously described (14). R1, R2, and R3 denote direct repeats of ≥90% sequence identity.

DSM 20016^T^ and ATCC PTA-6475 (Table 1) were resequenced using a hybrid ONT–Illumina approach (11). Materials and Methods are detailed in the Supplemental Material. Both assemblies produced 2.04 Mbp circular chromosomes with >99.7% identity to the previously published JCM 1112^T^ and ATCC PTA-6475 sequences (Table 2). Importantly, both regions absent from the previous DSM 20016^T^ closed genome sequence (2) were present in the DSM 20016^T^ sequence generated here (Fig. 1A). This is consistent with multiple DSM 20016^T^ sequencing efforts since 2007 (6–8, 12), including studies using stocks acquired directly from DSMZ, although this study is the first to produce another publicly available closed genome sequence.

**TABLE 1:** Strains used in this study

Strain (aliases)	Genotype	Characteristics	References
PRB50 (DSM 20016^T^, F 275^T^)	Wild type	Britton lab stock of DSM 20016^T^ used for whole-genome sequencing.	(1, 2)
PRB20 (ATCC PTA-6475, MM4-1A)	Wild type	Britton lab stock of ATCC PTA-6475 used for whole-genome sequencing.	(9, 10)
VPL1014 (ATCC PTA-6475, MM4-1A)	Wild type	van Pijkeren lab stock of ATCC PTA-6475 used for protein analysis.	(13)
VPL4359 (*cmbA*^−^)	VPL1014::*cat cmbA*^−^	VPL1014 with *cat* insertion (chloramphenicol resistance) and *cmbA* inactivation (P282*N283D) used for protein analysis.	(13)

**TABLE 2:** Sequencing and assembly statistics

Statistic	DSM 20016^T^	ATCC PTA-6475
ONT	SRR35954031	SRR35954029
Total sequenced bases (bp)	233,516,803	207,130,827
Total reads	62,782	62,843
Total reads *N*_50_ (bp)	5,778	5,195
Filtered sequenced bases (bp)	101,484,188	81,256,500
Filtered reads	10,006	8,110
Filtered reads *N*_50_ (bp)	10,320	10,203
Illumina	SRR35954030	SRR35954028
Total read pairs	7,623,028	15,630,276
Filtered read pairs	7,554,554	15,514,467
Assembly	GCF_054729175	GCF_054729215
Contig number	1	1
Genome size (bp)	2,041,522	2,040,655
Mean ONT depth (×)	50	40
Mean Illumina depth (×)	1,068	2,169
GC content (%)	38.89	38.88
Annotated genes	2,090	2,090
CDSs	1,999	1,999
Protein-coding genes	1,935	1,935
Pseudogenes	64	64
RNA genes	91	91
rRNA genes (5S, 16S, 23S)	18 (6, 6, 6)	18 (6, 6, 6)
tRNA genes	69	69
Non-coding RNA genes	4	4
CheckM completeness (%)	98.10	98.10
CheckM contamination (%)	0.72	0.72

To investigate the earlier discrepancy, we realigned the archived reads of the DSM 20016^T^ sequencing effort from 2007 to our assembly. The reads showed clear coverage within the 8.4 kbp region, but none across the 30.2 kbp region (Fig. S1). This suggests that, while the absence of the 8.4 kbp region was an assembly artifact, the 30.2 kbp region was genuinely deleted in the laboratory stock used for that previous sequencing. Nevertheless, our and others’ work has demonstrated that both regions are intact in many subcultures of DSM 20016^T^. Therefore, we want to emphasize that their absence should not be considered a founder mutation of DSM 20016^T^ and that the strain acquired directly from DSMZ should not be expected to lack these regions.

A complete list of sequence conflicts between the previous JCM 1112^T^ sequence and this study’s DSM 20016^T^ sequence is provided in Table S1, and conflicts between the previous ATCC PTA-6475 draft sequence and this study’s sequence are provided in Table S2. Besides conflicts in and around rRNA operons, the most substantial conflicts are in *cmbA*, located in the 30.2 kbp region. CmbA/Lar_0958 is a large cell-wall-anchored fibrillar adhesin that binds host mucus. It has a variable number of tandemly repeated ~100 aa domains, arranged like beads on a string (4, 5, 13). These protein repeats are in turn encoded by a stretch of perfectly identical ~300 bp tandem nucleotide repeats, thus composing an intragenic macrosatellite (15). Published sequences disagree on the number of nucleotide repeats between and within strains, with one report finding intrastrain variability in repeat number (2–5). In our assemblies, DSM 20016^T^ contains 11 repeats (3,165 bp) and ATCC PTA-6475 contains 8 repeats (2,301 bp) (Fig. 1B). The only other non-syntenic locus between the two strains is a reciprocal translocation between the specificity subunit genes of the type I restriction–modification system LreRMII (Fig. 1C), a rearrangement previously characterized by our lab (14).

The *cmbA* repeat counts determined here are notably higher than those previously published: five repeats in DSM 20016^T^/JCM 1112^T^ and one to three repeats in ATCC PTA-6475 (3, 4, 10). As the earlier assemblies used Sanger and short-read sequencing, which are known to systematically undercount tandem repeats (16), they likely underestimated the repeat count of *cmbA*.

**Fig 2:**
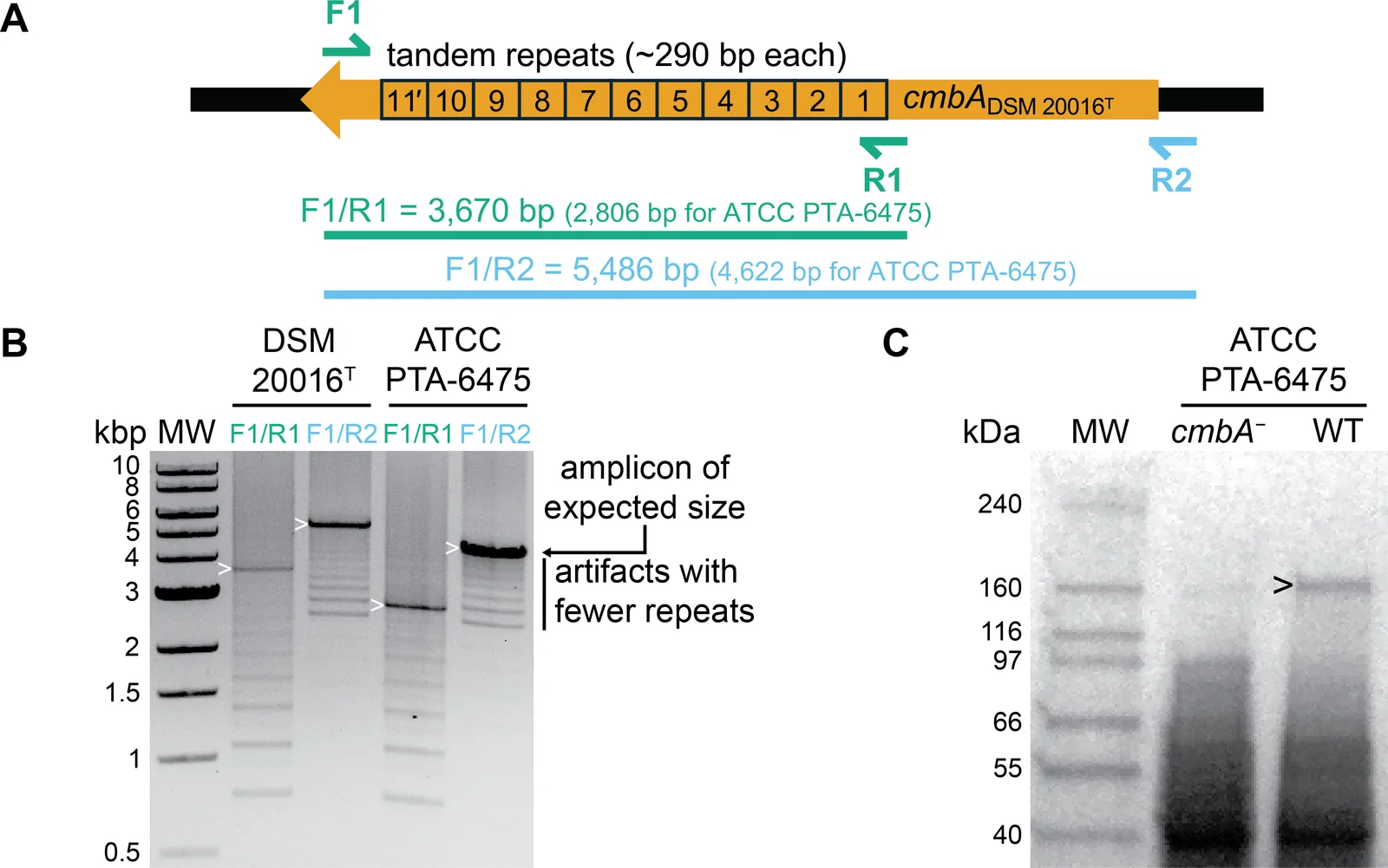
Tandem repeats in *cmbA* generate PCR artifacts. (**A**) Map of primers for PCR amplification of *cmbA*. See Table S3 for primer sequences. (**B**) Agarose gel of PCR products showing a strong band of the expected size (indicated by arrowheads) and a ladder corresponding to lower-molecular-weight artifacts with different numbers of repeats. (**C**) Coomassie-stained polyacrylamide gel of cell wall extracts from ATCC PTA-6475 *cmbA*^−^ (VPL4359) and wild type (VPL1014, “WT”), with the band matching the predicted molecular weight of mature CmbA indicated by the arrowhead. MW, molecular weight marker.

Based on our sequencing, *cmbA* is the longest coding sequence in both DSM 20016^T^ and ATCC PTA-6475, at 5,397 and 4,533 bp, respectively. We attempted to confirm these lengths with PCR using primers flanking the repeats (Fig. 2A, see Table S3 for primers). In addition to an amplicon of the size expected from our sequencing, agarose gel of PCR products consistently showed a ladder of smaller fragments (spaced by the length of one repeat) and a higher-molecular-weight smear (Fig. 2B). Prior work has shown that this pattern of chimeric amplicons arises when amplifying any stretch of long identical tandem repeats, which can lead to out-of-register annealing and polymerase slipping (17). Replacing the reverse primer (R1) with one which instead annealed 1.8 kbp downstream of the repeats (R2) led to an enrichment of the expected amplicon over the lower-molecular-weight ladder, although the ladder did not disappear completely (Fig. 2B). Purification of the target fragments, followed by ONT sequencing and *de novo* assembly, yielded sequences and repeat counts identical to those obtained from whole-genome sequencing for both strains (Fig. S2). These observations suggest that the earlier report of intrastrain variability in *cmbA* repeat number based on agarose gel of PCR products (5) was the result of PCR artifacts rather than genuine strain-level heterogeneity. The same artifacts, combined with the maximum read length of Sanger sequencing, may have also contributed to the previous report of only one repeat in ATCC PTA-6475 (4).

Finally, we investigated CmbA using polyacrylamide gel electrophoresis of cell wall extracts of ATCC PTA-6475, for which a CmbA knockout strain is available (13). From our sequencing, the eight-repeat version of CmbA present in ATCC PTA-6475 is expected to have a molecular weight of 155 kDa after processing. A single ~160 kDa band was present in the wild-type strain but absent in the *cmbA*-knockout derivative (Fig. 2C), consistent with our sequencing. The band was confirmed by mass spectrometry to be CmbA (of indeterminate repeat number), which was ~1,000× more abundant in the wild-type sample (Fig. S3). While the molecular weight by SDS-PAGE is consistent with the eight-repeat version determined by sequencing, a limitation of the approach is that, since the expected molecular weight of a single repeat is only 10 kDa, we cannot rule out strain-level variation at the level of one or a few repeats. Furthermore, if CmbA is post-translationally modified, as is common for cell-surface proteins, SDS-PAGE may overestimate the repeat number.

The large size and numerous repeats of CmbA raise interesting questions about the functional significance of repeat number. In MUB, another *L. reuteri* fibrillar adhesin with a similar “bead-on-a-string” organization, the tandem repeats directly mediate multivalent interactions with terminal sialic acid residues on host mucus (18). As anti-repeat antibodies reduce CmbA-mediated mucosal adhesion *in vitro* (5), we hypothesize that the tandem repeats in CmbA mediate adhesion similarly to MUB. Additional repeats could therefore strengthen mucosal adhesion, although the 8-repeat ATCC PTA-6475 and 11-repeat DSM 20016^T^ generally adhere equally well *in vitro* (5, 19, 20). *cmbA* mutants with varied repeat numbers and deleted domains will help in testing this hypothesis. We expect that the sequences reported here will facilitate further investigations into *L. reuteri* biology.

## Supplementary Material

Reviewer comments

## Data Availability

The sequencing data generated in this study have been deposited with NCBI under BioProject accession number PRJNA1357875 and BioSample accession numbers SAMN53091637 (DSM 20016^T^) and SAMN53091638 (ATCC PTA-6475). The assembled genome sequences have been deposited with GenBank under accession numbers JBTLXI010000001 (DSM 20016^T^) and JBTLXH010000001 (ATCC PTA-6475). The raw reads have been deposited under Sequence Read Archive accession numbers SRR35954031 (DSM 20016^T^, ONT), SRR35954030 (DSM 20016^T^, Illumina), SRR35954029 (ATCC PTA-6475, ONT), and SRR35954028 (ATCC PTA-6475, Illumina).

## SUPPLEMENTAL MATERIAL

Supplemental Material spectrum.04005-25-s0001.docx Supplemental materials and methods, Figures S1 to S3, and Tables S1 to S3.

OPEN PEER REVIEW reviewer-comments.pdf An accounting of the reviewer comments and feedback.

ASM does not own the copyrights to Supplemental Material that may be linked to, or accessed through, an article. The authors have granted ASM a non-exclusive, world-wide license to publish the Supplemental Material files. Please contact the corresponding author directly for reuse.
