# Supplementary material for: Revised complete genome sequences of Limosilactobacillus reuteri DSM 20016T and ATCC PTA-6475 and confirmation of an intragenic macrosatellite in adhesin gene cmbA
Source: Microbiol Spectr. 2026 Jun 15;14(7):e04005-25. doi: 10.1128/spectrum.04005-25 (PMC13339910; doi:10.1128/spectrum.04005-25)
Supplement: Supplemental Material — s and methods, Figures S1 to S3, and Tables S1 to S3. [file spectrum.04005-25-s0001.docx]

# Supplemental Materials and Methods

## Strains and sequencing

See Table 1 for a list of strains used in this study. For sequencing, the lab stocks PRB50 and PRB20 were used, which are subcultures of DSM 20016^T^ and ATCC PTA-6475, respectively. PRB50 derives from an agar stab of DSM 20016^T^ sent to our lab by Dr. Stefan Roos, Swedish University of Agricultural Sciences, in 2005. PRB20 is a frozen stock of ATCC PTA-6475 (original strain designation MM4-1A), sent to our lab by BioGaia in 2006. Frozen stocks were stored at −80 °C. For each strain, 15 mL of fresh sterile-filtered Lactobacillus MRS Broth (Research Products International) was inoculated with ~1 μL of frozen stock and incubated statically at 37 °C to an optical density at 600 nm of ~1 (GENYSYS 20, Thermo Scientific). Cells were pelleted at room temperature at 3000 × *g* for 10 min, washed once in 10 mL sterile phosphate-buffered saline, resuspended in 500 μL DNA/RNA Shield (Zymo Research), and stored at room temperature until sequencing.

Hybrid (ONT + Illumina) Bacterial Genome Sequencing with Extraction was performed by Plasmidsaurus (<https://plasmidsaurus.com/technical-documentation/genome>). See Table 2 for sequencing and assembly statistics. ONT was performed on an R10.4.1 flow cell with Dorado basecalling (dna_r10.4.1_e8.2_400bps_sup@v4.3.0 model). Basecalled ONT reads were filtered, assembled, and polished following a previously described workflow (1). ONT reads were filtered with Filtlong v0.3.1 (2) by discarding reads < 6 kbp, followed by the lowest-quality 10%. Filtered ONT reads were assembled with Trycycler v0.5.6 (3) using Flye v2.9.6 (4), miniasm v0.3 (5) with Minipolish v0.2.0 (6), and Raven v1.8.3 (7). The cluster of length closest to 2 Mbp (the approximate size of both genomes) was chosen for consensus generation. This produced single ~2.04 Mbp circular contigs for both genomes, which were polished with Medaka v2.1.1 (8). For Illumina, 150-bp paired-end reads were quality-filtered with fastp v1.0.1 (9) and used to further polish the ONT assembly with Polypolish v0.6.1 (10), yielding the final assemblies, which were annotated with NCBI Prokaryotic Genome Annotation Pipeline v6.10 (11).

Base changes introduced during polishing were inspected and validated manually against aligned reads with Integrative Genomics Viewer (IGV) v2.13.2 (12). Sequence conflicts between this work’s sequences and reference sequences (DSM 20016^T^, Frese et al. (13): [NC_009513.1](https://www.ncbi.nlm.nih.gov/nuccore/NC_009513.1); JCM 1112^T^, Morita et al. (14): [NC_010609.1](https://www.ncbi.nlm.nih.gov/nuccore/NC_010609.1), annotation GCF_000010005.1-RS_2026_01_20; DSM 20016^T^, Sun et al. (15): [NZ_AZDD01](https://www.ncbi.nlm.nih.gov/nuccore/951332068); ATCC PTA-6475, Saulnier et al. (16): [NZ_ACGX02](https://www.ncbi.nlm.nih.gov/nuccore/325683662), annotation GCF_000159475.2-RS_2025_12_07) were inspected with IGV, SnapGene v8.2.1 (Dotmatics), and NCBI BLAST (17). Statistics were computed with n50 v1.9.3 (18), SAMtools v1.22.1 (19), and CheckM v1.2 (20). Visualizations were prepared with Ziplign v1.0.1 (21), SVbyEye v0.99.0 (22), IGV, and SnapGene.

For the DSM 20016^T^ sequence published by Frese et al. (13), “Raw Sequence Data” were downloaded from JGI GOLD (<https://gold.jgi.doe.gov/projects?id=Gp0000135>) and processed with sff2fastq v0.9.2 (23).

## PCR

Primers were designed with Primer-BLAST (24) and ordered from Integrated DNA Technologies. See Table S3 for a list of primers used. Genomic DNA for PCR was prepared from DSM 20016^T^ and ATCC PTA-6475 with the DNeasy PowerLyzer Microbial Kit (Qiagen) following manufacturer instructions and quantified on a DS-11 spectrophotometer (DeNovix). For PCR, we attempted to optimize reaction conditions by modifying several parameters, including polymerase (*Taq* versus Q5), template quantity, primer pair, annealing temperature, extension duration, and cycle count. The final PCR reactions were prepared with Q5 Hot Start High-Fidelity 2X Master Mix (New England Biolabs) following manufacturer instructions for a reaction volume of 25 μL with 1 ng of genomic DNA as template. Final cycling conditions were 98 °C 30 s, (98 °C 10 s, 55 °C 30 s, 72 °C 3 min) × 30, 72 °C 2 min. 10 μL of PCR product was electrophoresed at 130 V for 1 h on a 1% agarose gel in Tris-acetate-EDTA with a 1-mm B1-10 comb (Thermo Fisher Scientific) alongside 1 kb DNA Ladder (New England Biolabs). The gel was imaged on an AlphaImager HP (ProteinSimple) using manufacturer presets and 277-ms exposure.

For ONT sequencing of the PCR products, reactions were purified with the MinElute PCR Purification Kit (Qiagen) and subjected to Standard Purified Linear/PCR ONT sequencing from Plasmidsaurus. Raw reads were filtered as described in the previous section, then assembled with Flye.

## Cell wall extraction and protein analysis

For protein gel analysis, we used a *cmbA*-knockout derivative of ATCC PTA-6475 (VPL4359) and its genetic-background control (VPL1014), gifts of Dr. Jan-Peter van Pijkeren, University of Wisconsin–Madison (25). Cell-wall extracts were prepared by mutanolysin solubilization as previously described (26). In brief, 10 mL of overnight culture was washed with phenylmethylsulfonyl fluoride, incubated with 10 mg/mL lysozyme and 250 U/mL mutanolysin at 37 °C for 2 h, and the supernatant collected. Protein was quantified by Pierce BCA Protein Assay (Thermo Fisher Scientific) and 20 μg of protein in Laemmli Sample Buffer (Bio-Rad Laboratories) + 2.5% β-mercaptoethanol was boiled for 5 min and electrophoresed at 200 V for 50 min in a 4–20% Mini-PROTEAN TGX Precast Protein Gel (Bio-Rad Laboratories) following manufacturer instructions alongside HiMark Unstained Protein Standard (Thermo Fisher Scientific). The gel was stained with Coomassie-based GelCode Blue Safe Protein Stain (Thermo Fisher Scientific) following manufacturer instructions and imaged on an AlphaImager HP using manufacturer presets and 51-ms exposure.

For mass spectrometry, a fresh gel was electrophoresed with one blank lane separating each sample. A ~0.5-cm^2^ ~160-kDa region was excised from each lane and stored at 4 °C until analysis by the Baylor College of Medicine Mass Spectrometry Proteomics Core. Samples were in-gel digested and run on an Orbitrap-based mass spectrometer with a CmbA-targeted method. Data were searched against the RefSeq protein database for *L. reuteri* MM4-1A (NCBI:txid548485) using Proteome Discoverer (Thermo Fisher Scientific).

**
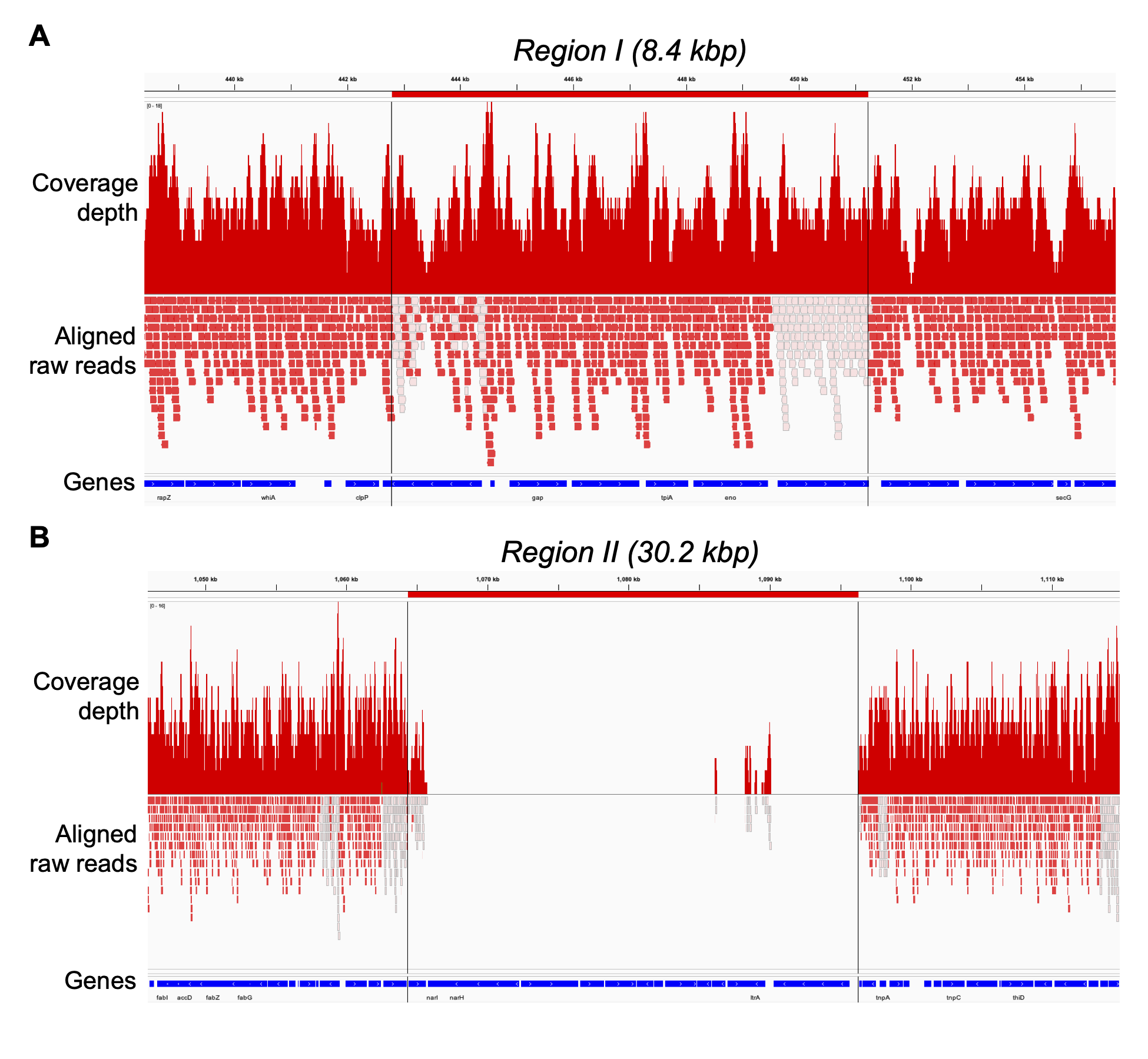
Fig S1** Archived reads from an earlier DSM 20016^T^ sequence suggest presence of Region I, but not Region II, in the previously sequenced isolate. (**A**) Alignment of the pyrosequencing reads from the DSM 20016^T^ sequence reported by Frese et al. (13) against the DSM 20016^T^ sequence generated in this study, indicating presence of an intact but unassembled Region I. (**B**) Conversely, Region II has no coverage. Regions I and II refer to the regions unique to JCM 1112^T^ compared to DSM 20016^T^ as previously described (14). Lighter shading within the aligned reads indicates multiply aligned reads, generally within multicopy mobile genetic elements. Visualized with IGV.

**
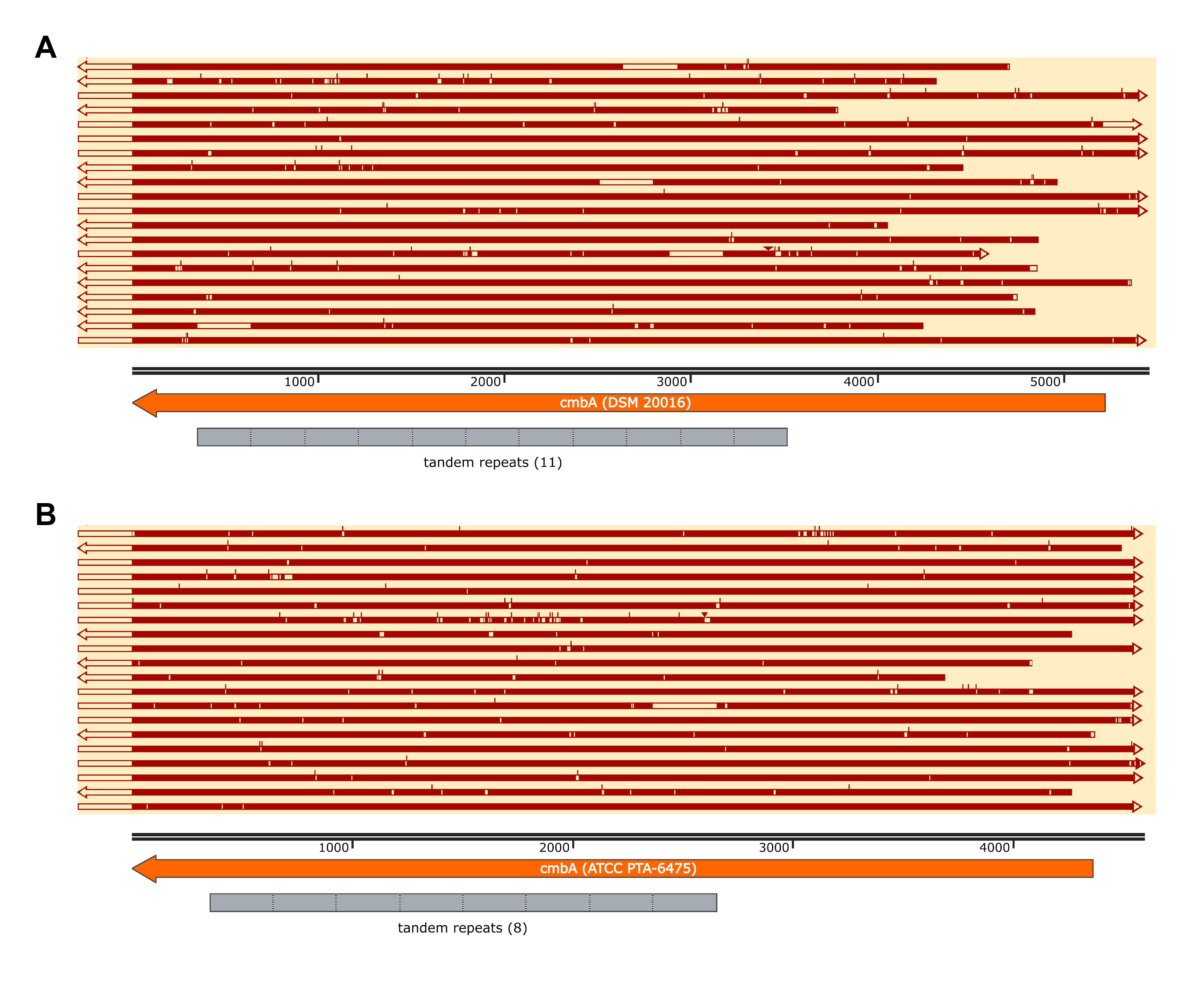
Fig S2** Targeted PCR of *cmbA* followed by ONT sequencing and *de novo* alignment yields the same sequences as whole-genome ONT sequencing. Amplicons of *cmbA* from DSM 20016^T^ (**A**) and ATCC PTA-6475 (**B**) were subjected to ONT sequencing, filtered for quality, and assembled *de novo* using Flye. A subset of 20 aligned reads is shown for each strain. Solid fill indicates alignment, empty fill indicates deletions, and ticks and arrowheads indicate insertions relative to the consensus. One repeat is occasionally omitted in reads from both strains. Visualized with SnapGene.

**
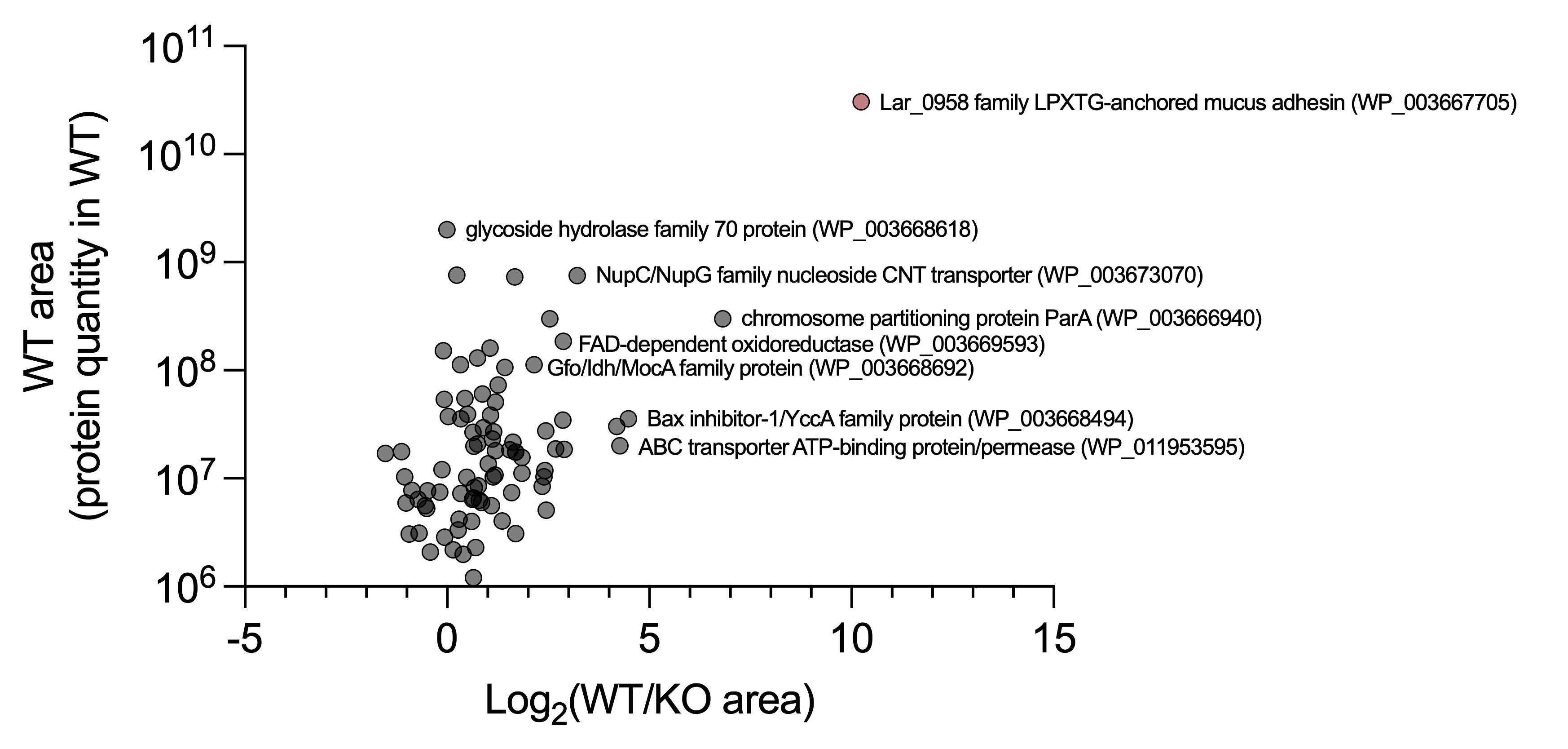
Fig S3** Positive identification of the excised band from Fig. 2C as CmbA. The ~160-kDa region from polyacrylamide gel electrophoresis of cell wall extracts of *L. reuteri* VPL1014 (wild type, WT) and VPL4359 (*cmbA*^−^, KO) were subjected to mass spectrometric analysis. Peptides were searched against the RefSeq proteome of *L. reuteri* MM4-1A and total peak area for each protein calculated. CmbA is marked in red. The glycoside hydrolase family 70 protein, with a molecular weight of 168 kDa, is the second most abundant protein in the WT sample, the most abundant protein in the KO sample, and the likely identity of the faint ~160-kDa band seen in the KO in Fig. 2C.

**TABLE S1** Summary of the sequence conflicts between the closed JCM 1112^T^ genome sequence ([NC_010609.1](https://www.ncbi.nlm.nih.gov/nuccore/NC_010609.1)) by Morita et al. (14) and the closed DSM 20016^T^ genome sequence generated as part of this work ([NZ_CM138830.1](https://www.ncbi.nlm.nih.gov/nuccore/NZ_CM138830.1))

| **JCM 1112^T^ →  DSM 20016^T^*^a^*** | **Intra- genic** | **JCM 1112^T^, Morita et al. (14)** | | **DSM 20016^T^, this work** | |
| --- | --- | --- | --- | --- | --- |
|  |  | **Position*^b^*** | **RefSeq locus tag (gene)** | **Position*^b^*** | **RefSeq locus tag (gene)** |
| A → G | + | 178,405 | LAR_RS00750 (16S rRNA) | 178,405 | ACX3VP_RS00765 (16S rRNA) |
| G → A | + | 178,509 |  | 178,509 |  |
| C → T | + | 178,624 |  | 178,624 |  |
| A → G | + | 178,634 |  | 178,634 |  |
| A → G | − | 178,968 | 5′ of LAR_RS00755 (tRNA-Ile) | 178,968 | 3′ of ACX3VP_RS00765  (16S rRNA) |
| C insertion | − | 178,976–7 |  | 178,977 |  |
| 201-bp  deletion | + | 178,979–9,179 | LAR_RS00755 (tRNA-Ile),  LAR_RS00760 (tRNA-Ala) | 178,979–80 |  |
| T → C | − | 179,184 | 3′ of LAR_RS00760  (tRNA-Ala) | 178,984 |  |
| C → A | + | 179,442 | LAR_RS00765 (23S rRNA) | 179,242 | ACX3VP_RS00770 (23S rRNA) |
| C deletion | − | 264,239 | 5′ of LAR_RS01190  (CTP synthase) | 264,038–9 | 5′ of ACX3VP_RS01200  (CTP synthase) |
| G → A  (K_7_ → K_7_) | + | 305,046 | LAR_RS01380 (*ftsH*) | 304,845 | ACX3VP_RS01390 (*ftsH*) |
| C insertion  (frameshift)*^c^* | + | 305,132–3 |  | 304,932 |  |
| A → G | + | 312,422 | LAR_RS01405 (16S rRNA) | 312,222 | ACX3VP_RS01415 (16S rRNA) |
| C → T | + | 312,428 |  | 312,228 |  |
| T → C | + | 312,501 |  | 312,301 |  |
| A → G | + | 313,270 |  | 313,070 |  |
| TATA deletion | + | 313,752–5 |  | 313,551–2 |  |
| T → C  (L_275_ → L_275_) | + | 441,164 | LAR_RS02035 (*whiA*) | 440,960 | ACX3VP_RS02050 (*whiA*) |
| A deletion  (frameshift)*^c^* | + | 454,863 | LAR_RS02090 (*secG*) | 454,658–9 | ACX3VP_RS02110 (*secG*) |
| T insertion  (frameshift)*^c^* | + | 497,458–9 | LAR_RS02325  (IS*L3* family transposase) | 497,254 | ACX3VP_RS02355  (IS*L3* family transposase) |
| T → C  (V_114_ → A_114_) | + | 537,244 | LAR_RS02535  (IS*Lre2*-like element  IS*Lre2* family transposase) | 537,040 | ACX3VP_RS02575  (IS*Lre2*-like element  IS*Lre2* family transposase) |
| C → T  (T_162_ → T_162_) | + | 537,389 |  | 537,185 |  |
| CTTGTGCAC  insertion  (L_190_V_191_H_192_) | + | 537,471–2 |  | 537,268–76 |  |
| A → C  (V_23_ → G_23_) | + | 562,967 | LAR_RS02655 (*rpsD*) | 562,772 | ACX3VP_RS02700 (*rpsD*) |
| A deletion | − | 590,266 | 3′ to LAR_RS02760 (*pgsA*) | 590,070–1 | 3′ to ACX3VP_RS02805 (*pgsA*) |
| G → A | + | 632,759 | LAR_RS02985 (16S rRNA) | 632,563 | ACX3VP_RS03030 (16S rRNA) |
| A → G | + | 632,895 |  | 632,699 |  |
| T → C | + | 632,974 |  | 632,778 |  |
| A → G | + | 633,743 |  | 633,547 |  |
| 248-bp  deletion | + | 634,225–472 | LAR_RS02985 (16S rRNA),  LAR_RS02990 (23S rRNA) | 634,028–9 | ACX3VP_RS03030 (16S rRNA),  ACX3VP_RS03035 (tRNA-Ile),  ACX3VP_RS03040 (tRNA-Ala),  ACX3VP_RS03045 (23S rRNA) |
| 444-bp  insertion | + | 634,472–3 |  | 634,029–472 |  |
| A → G | + | 635,160 | LAR_RS02990 (23S rRNA) | 635,160 | ACX3VP_RS03045 (23S rRNA) |
| C → T | + | 635,185 |  | 635,185 |  |
| T → C | + | 636,071 |  | 636,071 |  |
| C → T | + | 636,230 |  | 636,230 |  |
| G → A | – | 637,394 | 3′ of LAR_RS02990  (23S rRNA) | 637,394 | 3′ of ACX3VP_RS03045  (23S rRNA) |
| A deletion | – | 637,410 |  | 637,409–10 |  |
| G → A | + | 649,191 | LAR_RS03145 (16S rRNA) | 649,190 | ACX3VP_RS03200 (16S rRNA) |
| A → G | + | 649,327 |  | 649,326 |  |
| T → C | + | 649,406 |  | 649,405 |  |
| 248-bp  deletion | + | 650,657–904 | LAR_RS03145 (16S rRNA),  LAR_RS03150 (23S rRNA) | 650,655–6 | ACX3VP_RS03200 (16S rRNA),  ACX3VP_RS03205 (tRNA-Ile),  ACX3VP_RS03210 (tRNA-Ala),  ACX3VP_RS03215 (23S rRNA) |
| 444-bp  insertion | + | 650,904–5 |  | 650,656–1,099 |  |
| C → T | + | 651,544 | LAR_RS03150 (23S rRNA) | 651,749 | ACX3VP_RS03215 (23S rRNA) |
| A → G | + | 651,592 |  | 651,787 |  |
| T → C | + | 652,449 |  | 652,644 |  |
| G → C  (E_316_ → D_316_) | + | 762,165 | LAR_RS03730 (*ltrA*) | 762,360 | ACX3VP_RS03800 (*ltrA*) |
| G deletion (frameshift)*^c^* | + | 762,495 |  | 762,689–90 |  |
| A insertion  (frameshift)*^c^* | + | 840,202–3 | LAR_RS10970 (ECF  transporter S component), LAR_RS10975 (ECF  transporter S component) | 840,397 | ACX3VP_RS04165  (ECF transporter S component) |
| C → T  (C_730_ → Y_730_) | + | 946,293 | LAR_RS04685  (YfhO family protein) | 946,488 | ACX3VP_RS04790  (YfhO family protein) |
| C deletion  (frameshift)*^c^* | + | 956,306 | LAR_RS04725 (*sufC*) | 956,500–1 | ACX3VP_RS04835 (*sufC*) |
| G deletion  (frameshift)*^c^* | + | 962,761 | LAR_RS04760  (response regulator) | 962,954–5 | ACX3VP_RS04870  (response regulator) |
| T → C | − | 976,645 | 3′ of LAR_RS04825  (DUF1440 domain- containing protein) | 976,838 | 3′ of ACX3VP_RS04935  (DUF1440 domain- containing protein) |
| TAAAAG GGGGA  deletion | − | 993,474–84 | 3′ of LAR_RS04895  (THUMP domain-containing  class I SAM-dependent RNA  methyltransferase) | 993,666–7 | 3′ of ACX3VP_RS05010  (THUMP domain-containing  class I SAM-dependent RNA  methyltransferase) |
| G → T  (M_117_ → I_117_) | + | 1,031,437 | LAR_RS05080  (ATP-binding protein) | 1,031,619 | ACX3VP_RS05195  (ATP-binding protein) |
| C → T  (A_436_ → T_436_) | + | 1,058,039 | LAR_RS10590  (IS*L3* family transposase)*^d^* | 1,058,221 | ACX3VP_RS05405  (IS*L3* family transposase)*^d^* |
| A → T  (H_427_ → Q_427_) | + | 1,058,064 |  | 1,058,246 |  |
| G → A  (E_35_ → E_35_) | + | 1,064,350 | LAR_RS05310  (IS*L3* family transposase) | 1,064,532 | ACX3VP_RS05430  (IS*L3* family transposase) |
| 1,728-bp  insertion  (576 aa) | + | 1,092,083–4 | LAR_RS05440  (Lar_0958 family LPXTG- anchored mucus adhesin)*^e^* | 1,092,266–3,993 | ACX3VP_RS05560  (Lar_0958 family LPXTG- anchored mucus adhesin)*^c^* |
| G → A | + | 1,114,337 | LAR_RS05545 (23S rRNA) | 1,116,247 | ACX3VP_RS05670 (23S rRNA) |
| G → A | + | 1,115,669 |  | 1,117,579 |  |
| T → C | + | 1,116,471 |  | 1,118,381 |  |
| G → A | + | 1,116,603 |  | 1,118,513 |  |
| G → A | + | 1,117,027 |  | 1,118,937 |  |
| C → T | + | 1,117,780 | LAR_RS05550 (16S rRNA) | 1,119,690 | ACX3VP_RS05675 (16S rRNA) |
| G → A | + | 1,117,877 |  | 1,119,787 |  |
| T → C | + | 1,117,884 |  | 1,119,794 |  |
| A → T | + | 1,118,653 |  | 1,120,563 |  |
| T → C | + | 1,118,732 |  | 1,120,642 |  |
| G → T | + | 1,118,757 |  | 1,120,667 |  |
| G → A | + | 1,118,851 |  | 1,120,761 |  |
| C → T | + | 1,118,919 |  | 1,120,829 |  |
| A deletion | − | 1,197,193 | 5′ of LAR_RS05905 (*tnpA*) | 1,199,102–3 | 5′ of ACX3VP_RS06030 (*tnpA*) |
| C deletion  (frameshift)*^c^* | + | 1,321,110 | LAR_RS06615  (nucleotidyltransferase) | 1,323,018–9 | ACX3VP_RS06765  (nucleotidyltransferase) |
| T → C | + | 1,410,584 | LAR_RS07115 (23S rRNA) | 1,412,492 | ACX3VP_RS07270 (23S rRNA) |
| A → G | + | 1,411,084 |  | 1,412,992 |  |
| G → A | + | 1,411,709 |  | 1,413,617 |  |
| T → C | + | 1,411,941 |  | 1,413,849 |  |
| C → T | − | 1,412,747 | Between  LAR_RS07115 (23S rRNA)  and  LAR_RS07120 (16S rRNA) | 1,414,655 | 3′ of ACX3VP_RS07275  (tRNA-Ala) |
| G → A | − | 1,412,775 |  | 1,414,683 |  |
| 201-bp  insertion | − →  + | 1,412,779–80 |  | 1,414,688–888 | ACX3VP_RS07275 (tRNA-Ala),  ACX3VP_RS07280 (tRNA-Ile) |
| G deletion | − | 1,412,784 |  | 1,414,892–3 | 5′ of ACX3VP_RS07280  (tRNA-Ile) |
| A → G | − | 1,412,788 |  | 1,414,896 |  |
| C → T | − | 1,412,791 |  | 1,414,899 |  |
| T → C | + | 1,413,125 | LAR_RS07120 (16S rRNA) | 1,415,233 | ACX3VP_RS07285 (16S rRNA) |
| C → T | + | 1,413,250 |  | 1,415,358 |  |
| T → C | + | 1,413,354 |  | 1,415,462 |  |
| C → T | + | 1,413,653 |  | 1,415,761 |  |
| CG → TA | + | 1,413,933–4 |  | 1,416,041–2 |  |
| C → T | + | 1,414,109 |  | 1,416,217 |  |
| A → G | + | 1,414,123 |  | 1,416,231 |  |
| C → T | + | 1,414,205 |  | 1,416,313 |  |
| T insertion  (frameshift)*^c^* | + | 1,431,946–7 | LAR_RS07215  (DDE-type integrase/​transposase/​recombinase) | 1,434,055 | ACX3VP_RS07385  (IS*3* family transposase) |
| A deletion  (frameshift)*^c^* | + | 1,511,728 | LAR_RS10890  (TOTE conflict system  archaeo-eukaryotic primase  domain-containing protein) | 1,513,836–7 | ACX3VP_RS07775  (TOTE conflict system  archaeo-eukaryotic primase  domain-containing protein) |

***^a^*** Substitutions are denoted as *base in JCM 1112^T^* → *base in DSM 20016^T^*, with insertions and deletions occurring in the JCM 1112^T^ sequence to yield the DSM 20016^T^ sequence. Amino acid changes are denoted in parentheses as *amino acid in JCM 1112^T^* → *amino acid in DSM 20016^T^*, if applicable, with position in the coding sequence in subscript.

***^b^*** *Position* indicates the range of the nucleotides listed or the nucleotides flanking an insertion or deletion site.

***^c^*** These insertions and deletions “repair” putatively inactivating frameshifts in the JCM 1112^T^ sequence.

***^d^*** This transposase pseudogene has already been putatively frameshifted upstream of these conflicts in both sequences.

***^e^*** This is *cmbA*. See main text for details. **TABLE S2** Summary of the sequence conflicts between the ATCC PTA-6475 draft genome sequence ([NZ_ACGX02](https://www.ncbi.nlm.nih.gov/nuccore/325683662)) by Saulnier et al. (16) and the closed ATCC PTA-6475 genome sequence generated as part of this work ([NZ_CM138831.1](http://www.ncbi.nlm.nih.gov/nuccore/NZ_CM138831.1))

| **Draft →  this work*^a^*** | **Intra- genic** | **ATCC PTA-6475 draft, Saulnier et al. (16)** | | **ATCC PTA-6475, this work** | |
| --- | --- | --- | --- | --- | --- |
|  |  | **Position*^b^*** | **RefSeq locus tag (gene)** | **Position*^b^*** | **RefSeq locus tag (gene)** |
| T deletion | − | 7:853,338 | 3′ of HMPREF0536_RS10105  (ABC transporter permease) | 40,575–6 | 3′ of ACX3VO_RS00195  (ABC transporter permease) |
| T deletion | + | 4:134,899 | HMPREF0536_RS01560 (tRNA-Thr) | 317,028–9 | ACX3VO_RS01435  (tRNA-Thr) |
| TTA deletion  (I_67_) | + | 4:135,599–601 | HMPREF0536_RS01570 (*tnpB*) | 317,727–8 | ACX3VO_RS01445 (*tnpB*) |
| T insertion | − | 5:209,349–50 | 3′ of HMPREF0536_RS02645 (*atpE*) | 526,053 | 3′ of ACX3VO_RS02505  (*atpE*) |
| C deletion | + | 1:1,116 | HMPREF0536_RS00050  (tRNA-Met) | 638,301–2 | ACX3VO_RS03090  (tRNA-Met) |
| C → T  (L_98_ → L_98_) | + | 6:462,694 | HMPREF0536_RS11365 (*tnpC*) | 655,641 | ACX3VO_RS03225 (*tnpC*) |
| C → T | − | 6:355,367 | Between  HMPREF0536_RS05115 (*ltrA*) and  HMPREF0536_RS05110 (*tnpA*) | 762,968 | Between  ACX3VO_RS03800 (*ltrA*) and  ACX3VO_RS03805 (*tnpA*) |
| T insertion  (frameshift)*^c^* | + | 6:53,052–3 | HMPREF0536_RS03535  (IS*L3* family transposase) | 1,065,283–2 | ACX3VO_RS05430  (IS*L3* family transposase) |
| A → G | − | 6:28,322 | 3′ of HMPREF0536_RS03405  (Lar_0958 family LPXTG- anchored mucus adhesin)*^d^* | 1,090,014 | 3′ of ACX3VO_RS05560  (Lar_0958 family LPXTG- anchored mucus adhesin)*^d^* |
| 1,440-bp  insertion  (480 aa) | + | 6:27,500–1 | HMPREF0536_RS03405  (Lar_0958 family LPXTG- anchored mucus adhesin)*^d^* | 1,092,275–0,836 | ACX3VO_RS05560  (Lar_0958 family LPXTG- anchored mucus adhesin)*^d^* |
| C deletion  (frameshift)*^c^* | + | 6:6,442 | HMPREF0536_RS03310 (*tnpC*) | 1,113,334–3 | ACX3VO_RS05660 (*tnpC*) |
| G → A  (V_44_ → V_44_) | + | 6:6,432 |  | 1,113,343 |  |
| T deletion | − | 6:4,721 | 3′ of HMPREF0536_RS03305  (5S rRNA) | 1,115,054–3 | 3′ of ACX3VO_RS05665  (5S rRNA) |
| C → A | + | 6:2,331 | HMPREF0536_RS03300  (23S rRNA) | 1,117,443 | ACX3VO_RS05670  (23S rRNA) |
| T insertion | − | 3:6,348–9 | Between  HMPREF0536_RS00525 (*tnpA*)  and  HMPREF0536_RS00520  (peptidoglycan recognition  protein family protein) | 1,198,520 | Between  ACX3VO_RS06030 (*tnpA*)  and  ACX3VO_RS06035  (peptidoglycan recognition  protein family protein) |
| A deletion | − | 7:114 | 3′ of HMPREF0536_RS05720  (hypothetical protein) | 1,228,014–5 | 3′ of ACX3VO_RS06200  (hypothetical protein) |
| T insertion | − | 7:1,244–5 | 5′ of HMPREF0536_RS05720  (hypothetical protein) | 1,229,145 | 5′ of ACX3VO_RS06200  (hypothetical protein) |
| A insertion | − | 7:1,293–4 |  | 1,229,195 |  |
| T insertion  (stop → stop) | + | 7:160,934–5 | HMPREF0536_RS06630  (amino acid permease) | 1,388,837 | ACX3VO_RS07125  (amino acid permease) |

***^a^*** Substitutions are denoted as *base in draft sequence* → *base in this work’s sequence*, with insertions and deletions occurring in the draft sequence to yield this work’s sequence. Amino acid changes are denoted in parentheses as *animo acid in draft sequence* → *amino acid in this work’s sequence*, if applicable, with position in the coding sequence in subscript.

***^b^*** *Position* indicates the range of the nucleotides listed or the nucleotides flanking an insertion or deletion site. For the draft sequence, positions are denoted as *contig*:*position*. Ranges in reverse numerical order denote reverse complementation.

***^c^*** These insertions and deletions “repair” putatively inactivating frameshifts in the draft sequence.

***^d^*** This is *cmbA*. See main text for details.

| **Name** | **Sequence (5′ → 3′)** | **Notes** |
| --- | --- | --- |
| F1 | CCAAAGCCGTTTTCAGCTCC | Amplify the direct repeats of *cmbA* with relatively short flanks on each end*.* |
| R1 | CTTCCAAAGCCACTAGCCCA |  |
| R2 | CGGCGGTCTCGCTACTAATC | Amplifies 2 kbp downstream of the direct repeats of *cmbA*. |

**TABLE S3** List of primers used in this study

**Supplementary References**

1. Wick RR, Judd LM, Holt KE. 2023. Assembling the perfect bacterial genome using Oxford Nanopore and Illumina sequencing. PLoS Comput Biol 19:e1010905.

2. Wick RR. 2025. Filtlong. <https://github.com/rrwick/Filtlong>. Retrieved 31 October 2025.

3. Wick RR, Judd LM, Cerdeira LT, Hawkey J, Méric G, Vezina B, Wyres KL, Holt KE. 2021. Trycycler: consensus long-read assemblies for bacterial genomes. Genome Biol 22:266.

4. Kolmogorov M, Yuan J, Lin Y, Pevzner PA. 2019. Assembly of long, error-prone reads using repeat graphs. Nat Biotechnol 37:540–546.

5. Li H. 2016. Minimap and miniasm: fast mapping and de novo assembly for noisy long sequences. Bioinformatics 32:2103–2110.

6. Wick RR, Holt KE. 2021. Benchmarking of long-read assemblers for prokaryote whole genome sequencing. F1000Res 8:2138.

7. Vaser R, Šikić M. 2021. Time- and memory-efficient genome assembly with Raven. Nat Comput Sci 1:332–336.

8. Oxford Nanopore Technologies. 2025. medaka. <https://github.com/nanoporetech/medaka>. Retrieved 31 October 2025.

9. Chen S. 2025. fastp 1.0: An ultra-fast all-round tool for FASTQ data quality control and preprocessing. iMeta 4:e70078.

10. Bouras G, Judd LM, Edwards RA, Vreugde S, Stinear TP, Wick RR. 2024. How low can you go? Short-read polishing of Oxford Nanopore bacterial genome assemblies. Microb Genomics 10:001254.

11. Tatusova T, DiCuccio M, Badretdin A, Chetvernin V, Nawrocki EP, Zaslavsky L, Lomsadze A, Pruitt KD, Borodovsky M, Ostell J. 2016. NCBI prokaryotic genome annotation pipeline. Nucleic Acids Res 44:6614–6624.

12. Robinson JT, Thorvaldsdóttir H, Wenger AM, Zehir A, Mesirov JP. 2017. Variant Review with the Integrative Genomics Viewer. Cancer Res 77:e31–e34.

13. Frese SA, Benson AK, Tannock GW, Loach DM, Kim J, Zhang M, Oh PL, Heng NCK, Patil PB, Juge N, Mackenzie DA, Pearson BM, Lapidus A, Dalin E, Tice H, Goltsman E, Land M, Hauser L, Ivanova N, Kyrpides NC, Walter J. 2011. The Evolution of Host Specialization in the Vertebrate Gut Symbiont *Lactobacillus reuteri*. PLoS Genet 7:e1001314.

14. Morita H, Toh H, Fukuda S, Horikawa H, Oshima K, Suzuki T, Murakami M, Hisamatsu S, Kato Y, Takizawa T, Fukuoka H, Yoshimura T, Itoh K, O’Sullivan DJ, McKay LL, Ohno H, Kikuchi J, Masaoka T, Hattori M. 2008. Comparative Genome Analysis of *Lactobacillus reuteri* and *Lactobacillus fermentum* Reveal a Genomic Island for Reuterin and Cobalamin Production. DNA Res 15:151–161.

15. Sun Z, Harris HMB, McCann A, Guo C, Argimón S, Zhang W, Yang X, Jeffery IB, Cooney JC, Kagawa TF, Liu W, Song Y, Salvetti E, Wrobel A, Rasinkangas P, Parkhill J, Rea MC, O’Sullivan O, Ritari J, Douillard FP, Paul Ross R, Yang R, Briner AE, Felis GE, de Vos WM, Barrangou R, Klaenhammer TR, Caufield PW, Cui Y, Zhang H, O’Toole PW. 2015. Expanding the biotechnology potential of lactobacilli through comparative genomics of 213 strains and associated genera. Nat Commun 6:8322.

16. Saulnier DM, Santos F, Roos S, Mistretta T-A, Spinler JK, Molenaar D, Teusink B, Versalovic J. 2011. Exploring Metabolic Pathway Reconstruction and Genome-Wide Expression Profiling in *Lactobacillus reuteri* to Define Functional Probiotic Features. PLoS ONE 6:e18783.

17. Altschul SF, Gish W, Miller W, Myers EW, Lipman DJ. 1990. Basic Local Alignment Search Tool. J Mol Biol 215:403–410.

18. Telatin A. 2025. n50. <https://github.com/quadram-institute-bioscience/n50>. Retrieved 31 October 2025.

19. Danecek P, Bonfield JK, Liddle J, Marshall J, Ohan V, Pollard MO, Whitwham A, Keane T, McCarthy SA, Davies RM, Li H. 2021. Twelve years of SAMtools and BCFtools. GigaScience 10:giab008.

20. Parks DH, Imelfort M, Skennerton CT, Hugenholtz P, Tyson GW. 2015. CheckM: assessing the quality of microbial genomes recovered from isolates, single cells, and metagenomes. Genome Res 25:1043–1055.

21. Hunt M. 2025. ziplign. <https://github.com/martinghunt/ziplign>. Retrieved 31 October 2025.

22. Porubsky D, Guitart X, Yoo D, Dishuck PC, Harvey WT, Eichler EE. 2025. SVbyEye: a visual tool to characterize structural variation among whole-genome assemblies. Bioinformatics 41:btaf332.

23. Das I. 2025. sff2fastq. <https://github.com/indraniel/sff2fastq>. Retrieved 5 November 2025.

24. Ye J, Coulouris G, Zaretskaya I, Cutcutache I, Rozen S, Madden TL. 2012. Primer-BLAST: A tool to design target-specific primers for polymerase chain reaction. BMC Bioinformatics 13:134.

25. Alexander LM, Khalid S, Gallego-Lopez GM, Astmann TJ, Oh J-H, Heggen M, Huss P, Fisher R, Mukherjee A, Raman S, Choi IY, Smith MN, Rogers CJ, Epperly MW, Knoll LJ, Greenberger JS, van Pijkeren J-P. 2024. Development of a *Limosilactobacillus reuteri* therapeutic delivery platform with reduced colonization potential. Appl Environ Microbiol 90:e00312-24.

26. Cole JN, Djordjevic SP, Walker MJ. 2008. Isolation and Solubilization of Gram-Positive Bacterial Cell Wall-Associated Proteins, p. 295–311. *In* Posch, A (ed.), 2D PAGE: Sample Preparation and Fractionation. Humana Press, Totowa, NJ.
