# Supplementary material for: Revised complete genome sequences of Limosilactobacillus reuteri DSM 20016T and ATCC PTA-6475 and confirmation of an intragenic macrosatellite in adhesin gene cmbA
Source: Microbiol Spectr. 2026 Jun 15;14(7):e04005-25. doi: 10.1128/spectrum.04005-25 (PMC13339910; doi:10.1128/spectrum.04005-25)
Supplement: Reviewer comments [file reviewer-comments.pdf]

AMERICAN SOCIETY FOR MICROBIOLOGY | Microbiology Spectrum

Revised complete genome sequences of *Limosilactobacillus reuteri* DSM 20016^T and ATCC PTA-6475 and confirmation of an intragenic macrosatellite in adhesin gene *cmbA*

Mohamed Kady and Robert Britton

Corresponding Author(s): Robert Britton, Baylor College of Medicine

Review Timeline:

Submission Date:	January 27, 2026
Editorial Decision:	April 13, 2026
Revision Received:	April 22, 2026
Accepted:	May 11, 2026

Editor: Steven Frese

Reviewer(s): The reviewers have opted to remain anonymous.

Transaction Report:

(Note: With the exception of the correction of typographical or spelling errors that could be a source of ambiguity, letters and reports are not edited. The original formatting of letters and referee reports may not be reflected in this compilation.)

DOI: https://doi.org/10.1128/spectrum.04005-25

Re: Spectrum04005-25 (Revised complete genome sequence of *Limosilactobacillus reuteri* DSM 20016^T and confirmation of an intragenic macrosatellite in adhesin gene *cmbA*)

Dear Dr. Robert A Britton:

Thank you for the privilege of reviewing your work. Below you will find my comments, instructions from the Spectrum editorial office, and the reviewer comments.

Please return the manuscript within 60 days; if you cannot complete the modification within this time period, please contact me. If you do not wish to modify the manuscript and prefer to submit it to another journal, notify me immediately so that the manuscript may be formally withdrawn from consideration by Spectrum.

Revision Guidelines
To submit your modified manuscript, log into the submission site at https://spectrum.msubmit.net/cgi-bin/main.plex. Go to Author Tasks and click the appropriate manuscript title to begin. The information you entered when you first submitted the paper will be displayed; update this as necessary. Note the following requirements:

• Upload point-by-point responses to the issues raised by the reviewers in a file named "Response to Reviewers," NOT in your cover letter.
• Upload a compare copy of the manuscript (without figures) as a "Marked-Up Manuscript" file.
• Upload a clean .DOC/.DOCX version of the revised manuscript and remove the previous version.
• Each figure must be uploaded as a separate, editable, high-resolution file (TIFF or EPS preferred), and any multipanel figures must be assembled into one file.
• Any supplemental material intended for posting by ASM should be uploaded with their legends separate from the main manuscript. You can combine all supplemental material into one file (preferred) or split it into a maximum of 10 files with all associated legends included.

For complete guidelines on revision requirements, see our Submission and Review Process webpage. Submission of a paper that does not conform to guidelines may delay acceptance of your manuscript.

Data availability: ASM policy requires that data be available to the public upon online posting of the article, so please verify all links to sequence records, if present, and make sure that each number retrieves the full record of the data. If a new accession number is not linked or a link is broken, provide Spectrum production staff with the correct URL for the record. If the accession numbers for new data are not publicly accessible before the expected online posting of the article, publication may be delayed; please contact production staff (Spectrum@asmusa.org) immediately with the expected release date.

Publication Fees: For information on publication fees and which article types are subject to charges, visit our website. If your manuscript is accepted for publication and any fees apply, you will be contacted separately about payment during the production process; please follow the instructions in that e-mail. Arrangements for payment must be made before your article is published.

ASM Membership: Corresponding authors may join or renew ASM membership to obtain discounts on publication fees. Need to upgrade your membership level? Please contact Customer Service at Service@asmusa.org.

The ASM Journals program strives for constant improvement in our submission and publication process. Please tell us how we can improve your experience by taking this quick Author Survey.

Thank you for submitting your paper to Spectrum.

Sincerely,
Steven Frese
Editor
Microbiology Spectrum

Reviewer #1 (Comments for the Author):

This short publication reports new complete genomes from two Limosilactobacillus reuteri strains, elucidating previously-reported discrepancies in genome sequences, and revealing the extent of repeats of an important multi-repeat adhesin (cmbA), as also confirmed by PCR and by SDS-PAGE analyses. This work has important implications for mechanistic studies of L. reuteri but also of multi-repeat proteins across bacteria, and highlight the need for careful and critical examination of existing genomes.

Minor comments as below;
The title may be revised to encompass the two L. reuteri strains for which the authors generated a novel closed genome sequence.
There seems to be an issue with reference numbering. There are two #7 in the reference list (Sun et al; Joergensen et al), and two #17 Connolly et al; Hommelsteim et al). I believe Joergensen should be #8, and Connolly, #9?

Reviewer #2 (Comments for the Author):

The study reported by Kady et al. is well justified, and the results are scientifically sound. It provides updated genome sequencing data for the type strain Limosilactobacillus reuteri DSM 20016 and strain MM4-1A, which is of clear practical value. The manuscript is well written, but requiring minor revisions in language and spelling.

Line 126: Consistent with this model... This sentence has clear grammatical errors, please correct.

Comments on Line 132-140:
It should be noted that SDS-PAGE generally lacks sufficient resolution to resolve proteins differing only in repeat number. In addition, conventional bottom-up mass spectrometry identifies peptides rather than intact proteins, making it difficult to determine the exact number of tandem repeats unless top-down MS is performed. The combined protein- and sequence-based validation approach assumes that all repeat-containing regions are fully preserved during transcription and translation. However, for surface proteins such as CmbA, post-translational modifications like glycosylation are common and could significantly alter the apparent molecular weight, which limits the reliability of inferring nucleotide-level repeat length from protein size.

Accurate determination of copy number of tandem repeats is still challenging for both sequencing technologies and genome-assembly algorithms. A more reliable strategy would be to avoid relying on the genome assemblers and instead map reads directly to the draft genome or a validated reference to estimate exact copy number. In addition, since the target fragment has already been amplified using the F1+R2 primer pair, it would be worthwhile to purify this fragment and subject it to ONT sequencing, or to design consecutive primers for full-length Sanger sequencing across the region to provide independent validation.

Figure 1: The label DSM1112 in panels B and C seems to be incorrect. Please double-check.

Response to Reviewers

Current manuscript title: Revised complete genome sequences of *Limosilactobacillus reuteri* DSM 20016^T and ATCC PTA-6475 and confirmation of an intragenic macrosatellite in adhesin gene *cmbA*

Manuscript number: Spectrum04005-25

We thank the reviewers for their feedback, which we have incorporated into the revised manuscript. Point-by-point responses follow. Changes from the original text are highlighted in red.

<u>Reviewer 1</u>

1. "The title may be revised to encompass the two L. reuteri strains for which the authors generated a novel closed genome sequence."

   The title has been updated to "Revised complete genome sequences of *Limosilactobacillus reuteri* DSM 20016^T and ATCC PTA-6475 and confirmation of an intragenic macrosatellite in adhesin gene *cmbA*."

2. "There seems to be an issue with reference numbering. There are two #7 in the reference list (Sun et al; Joergensen et al), and two #17 Connolly et al; Hommelsteim et al). I believe Joergensen should be #8, and Connolly, #9?"

   This has been corrected and all references have been double-checked for accuracy. We apologize for the confusion.

<u>Reviewer 2</u>

1. "Line 126: Consistent with this model... This sentence has clear grammatical errors, please correct."

   The sentence has been rewritten and now reads: "Replacing the reverse primer (R1) with one that annealed 1.8 kbp downstream of the repeats (R2) led to a relative enrichment of the expected amplicon versus the lower-molecular-weight ladder, although the ladder did not disappear completely."

2. "Comments on Line 132-140:

   "It should be noted that SDS-PAGE generally lacks sufficient resolution to resolve proteins differing only in repeat number. In addition, conventional bottom-up mass spectrometry identifies peptides rather than intact proteins, making it difficult to determine the exact number of tandem repeats unless top-down MS is performed. The combined protein- and sequence-based validation approach assumes that all repeat-containing regions are fully preserved during transcription and translation. However, for surface proteins such as CmbA, post-translational modifications like

glycosylation are common and could significantly alter the apparent molecular weight, which limits the reliability of inferring nucleotide-level repeat length from protein size."

The discussion in this paragraph has been rewritten to better emphasize the limitations of the SDS-PAGE and MS approaches. The relevant portion of the revised manuscript is: "The band was confirmed by mass spectrometry to be CmbA (of indeterminate repeat number), which was ~1,000× more abundant in the wild-type sample (Fig. S3). While the molecular weight by SDS-PAGE is consistent with the 8-repeat version determined by sequencing, a limitation of the approach is that, since the expected molecular weight of a single repeat is only 10 kDa, we cannot rule out strain-level variation at the level of one or a few repeats. Furthermore, if CmbA is post-translationally modified, as is common for cell-surface proteins, SDS-PAGE may overestimate the repeat number."

3. "Accurate determination of copy number of tandem repeats is still challenging for both sequencing technologies and genome-assembly algorithms. A more reliable strategy would be to avoid relying on the genome assemblers and instead map reads directly to the draft genome or a validated reference to estimate exact copy number. In addition, since the target fragment has already been amplified using the F1+R2 primer pair, it would be worthwhile to purify this fragment and subject it to ONT sequencing, or to design consecutive primers for full-length Sanger sequencing across the region to provide independent validation."

   As suggested, we have sequenced the PCR amplicons with ONT, *de novo* assembly of which yielded the same repeat counts. A sentence has been added to the results: "Purification of the target fragments, followed by ONT sequencing and *de novo* assembly, yielded sequences and repeat counts identical to those obtained from whole-genome sequencing for both strains (Fig. S2)." The new Figure S2 depicts alignment of raw reads from both strains. A relevant passage has been added to the supplemental methods.

4. "Figure 1: The label DSM1112 in panels B and C seems to be incorrect. Please double-check."

   The label was intended to read "DSM 20016." This has been fixed and all labels have been double-checked for accuracy. We apologize for the confusion.

We have also made a handful of other clarifying edits and fixed some accession numbers. These are all visible in the change-tracked version of the revised manuscript.

Re: Spectrum04005-25R1 (Revised complete genome sequences of *Limosilactobacillus reuteri* DSM 20016^{T} and ATCC PTA-6475 and confirmation of an intragenic macrosatellite in adhesin gene *cmbA*)

Dear Dr. Robert A Britton:

Your manuscript has been accepted, and I am forwarding it to the ASM production staff for publication. Your paper will first be checked to make sure all elements meet the technical requirements. ASM staff will contact you if anything needs to be revised before copyediting and production can begin. Otherwise, you will be notified when your proofs are ready to be viewed.

Data Availability: ASM policy requires that data be available to the public upon online posting of the article, so please verify all links to sequence records, if present, and make sure that each number retrieves the full record of the data. If a new accession number is not linked or a link is broken, provide production staff with the correct URL for the record. If the accession numbers for new data are not publicly accessible before the expected online posting of the article, publication may be delayed; please contact ASM production staff immediately with the expected release date.

Publication Fees: For information on publication fees and which article types have charges, please visit our website. We have partnered with Copyright Clearance Center (CCC) to collect author charges. If fees apply to your paper, you will receive a message from no-reply@copyright.com with further instructions. For questions related to paying charges through RightsLink, please contact CCC at ASM_Support@copyright.com or toll free at +1-877-622-5543. CCC makes every attempt to respond to all emails within 24 hours.

ASM Membership: Corresponding authors may join or renew ASM membership to obtain discounts on publication fees. Need to upgrade your membership level? Please contact Customer Service at Service@asmusa.org.

Embargo Policy: A press release may be issued as soon as the manuscript is posted on the Spectrum Latest Articles webpage. The corresponding author will receive an email with the subject line "ASM Journals Author Services Notification" when the article is available online.

The ASM Journals program strives for constant improvement in our submission and publication process. Please tell us how we can improve your experience by taking this quick Author Survey.

Thank you for submitting your paper to Spectrum.

Sincerely,
Steven Frese
Editor
Microbiology Spectrum
